# Evaluation of *Mycobacterium tuberculosis* viability in OMNIgene-SPUTUM reagent upon multi-day transport at ambient temperature

**DOI:** 10.1186/s12879-017-2756-3

**Published:** 2017-10-02

**Authors:** Elisa Tagliani, Riccardo Alagna, Silva Tafaj, Hasan Hafizi, Daniela Maria Cirillo

**Affiliations:** 1 Emerging Bacterial Pathogens Unit, Division of Immunology, Transplantation and Infectious Diseases, IRCCS San Raffaele Scientific Institute, Olgettina 58, 20132 Milan, Italy; 2 University Hospital Shefqet Ndroqi, Tirana, Albania

**Keywords:** *Mycobacterium tuberculosis*, Sample transport, Liquid culture, OMNIgene-SPUTUM

## Abstract

**Background:**

Maintaining the quality of clinical specimens for tuberculosis (TB) testing is a major challenge in many high TB burden-limited resources countries. Sample referral systems in low and middle income countries are often weak and the maintenance of the cold-chain challenging and very costly for TB programs. The development of transport media allowing the preservation of samples without refrigeration is critical for increasing access to TB diagnostic services and for reducing the costs related to testing.

**Methods:**

We evaluated the performance of OMNIgene-SPUTUM (OM-S) reagent for the maintenance of *Mycobacterium tuberculosis* (MTB) viability in sputum samples in the absence of refrigeration and its capacity to stabilize nucleic acid for molecular testing. A total of 329 sputum specimens from presumptive TB cases collected at the National Reference Laboratory in Tirana, Albania, were either decontaminated by a conventional method or processed with OM-S reagent and stored at room temperature. Samples in OM-S were shipped to the Supranational Reference Laboratory in Milan, Italy, at various times and processed for liquid culture.

**Results:**

Our data show that OM-S maintains MTB viability for at least three weeks in the absence of refrigeration and improves the quality of culture resulting in a contamination rate lower than 0.5%. However, a significant delay in the time to culture positivity was observed for samples stored for more than two weeks in OM-S.

**Conclusions:**

Overall, OM-S offers multiple benefits both at laboratory and TB national program level by increasing the availability to quality diagnostics, promoting access to health care services and strengthening TB patient care especially in hard to reach populations.

## Introduction

Despite great advances in tuberculosis (TB) diagnostics, a point of care testing for TB is yet not available and most of the procedures currently endorsed by the World Health Organization (WHO) including culture, phenotypic drug susceptibility testing (DST) and line probe assays (LPAs) are intended to be performed in higher level facilities and reference laboratories [1]. This requires the appropriate storage of the specimens at the collection site and their rapid transportation to the testing facility in temperature controlled conditions. The development of an efficient and reliable specimen referral system still represents a major challenge and cost burden for many TB programs worldwide. Several hurdles need to be overcome including the preservation of the quality and the integrity of the specimens during their referral to the testing site as well as the maintenance of the cold-chain infrastructure. The development of pre-analytical tools that allow the preservation of clinical specimens at ambient temperature and compatible with both conventional and molecular TB diagnostics may have multiple benefits including an increased access to TB testing, an improved quality of the testing and the shortening of the turn-around-time (TAT) and time to treatment. Additional potential benefits may include their use in drug resistance surveys where samples need to be collected also from remote and hard to reach areas and where logistic problems related to sample storage and transportation often affect the quality of the survey results. Similarly, the use of reagents for the long-term preservation and decontamination of specimens may greatly facilitate the process of international shipping of infectious clinical specimens while at the same time improving the quality of samples and TB testing.

DNA Genotek Inc. (Ottawa, Canada) has recently developed OMNIgene-SPUTUM (OM-S), a non-toxic sample transport reagent that eliminates the need for cold-chain infrastructure during sample transport. According to the company, this reagent liquefies and decontaminates sputum samples while at the same time preserving MTB viability for at least 8 days at ambient temperature and up to 40° [2]. Recent studies have shown OM-S compatibility with Xpert®MTB/RIF assay [3] and other routine TB tests [4].

We carried out a prospective study to evaluate the effectiveness of OM-S for the recovery of viable MTB bacteria in sputum samples stored at ambient temperature for different period of times and secondly, the capacity of OM-S to stabilize nucleic acid for LPA testing.

## Methods

### Clinical specimen collection and processing

This study was conducted as a collaboration between the TB National Reference Laboratory (NRL) in Tirana, Albania and the TB Supranational Reference Laboratory (SRL) in Milan, Italy, where the former was responsible for the collection, decontamination and liquid culture of the specimens and their shipment to Italy and the latter for the processing and culture of the OM-S treated samples and data analysis.

A total of 329 consecutive sputum specimens from TB presumptive cases were collected at NRL Tirana. Each specimen was first assessed by smear microscopy and categorized as negative or as positive with the following grading: scanty, 1+, 2+, 3+. Each sample was divided into two portions of approximately equal volume and randomly assigned to one treatment method. One aliquot was processed for liquid culture at the NRL Tirana on the same day of collection using the standard N-acetyl L-cysteine (NALC)/NaOH decontamination method, as previously described [5]. Decontaminated sediments were resuspended in1.5 ml of phosphate buffered saline and 0.5 ml inoculated into a Bactec MGIT 960 tubes. Samples were incubated at 37 °C for up to 41 days. The second aliquot was processed with OM-S reagent according to manufacturer’s instructions [6]. Briefly, the specimen was mixed with an equal volume of OM-S vortexed for 15–20 s and stored at ambient temperature at NRL Tirana. Samples in OM-S were shipped at ambient temperature to the TB SRL Milan, Italy on a weekly or bi-weekly base. Upon arrival at the SRL Milan, OM-S was removed by centrifugation at 2800 g for 20 min, the sediment was resuspended in 1.5 ml of sterile water and 0.5 ml of the suspension was inoculated into a Bactec MGIT 960 tube. Samples were incubated at 37 °C for up to 56 days using an extended MGIT protocol.

### GenoType MTBDRplus assay

Genomic DNA was extracted from 0.5 ml of OM-S decontaminated sediment using the commercially available kit GenoLyse (Hain Lifescience, Nehren, Germany). Only the samples from TB confirmed cases (i.e. culture positive for MTB) were processed for LPA (GenoType MTBDR*plus*; Hain Lifescience) testing. DNA amplification and hybridization were performed according to manufacturer’s instructions.

### Data collection and statistical analysis

The following variables were recorded for all samples included in the study: smear microscopy result including grade, liquid culture result including time to culture positivity -TPP- (i.e. number of days from sample inoculation to liquid culture positivity - days; hours-), date of sample collection at NRL Tirana, date of arrival and date of processing at SRL Milan, total number of days in OM-S reagent. Results were analyzed with Prism 4.0 (GraphPad Software). All comparisons were performed using a two-tailed Mann-Whitney U test. A *p*-value <0.05 was taken as statistically significant.

## Results

### Viability of MTB in OM-S reagent

A total of 329 consecutive sputum specimens from TB presumptive cases collected at NRL Tirana under routine conditions were included in this study. These comprised 48 smear positive specimens (11 scanty, 15 grade 1+, 6 grade 2+ and 16 grade 3+) and 281 smear negative. Following smear microscopy each sample was divided into two portions of equal volume and randomly assigned to one treatment method, namely standard NALC/NaOH decontamination or processing with OM-S reagent. While samples decontaminated by NALC/NaOH were directly inoculated into Bactec MGIT 960 tubes, the ones treated with OM-S were stored at ambient temperature for various times until shipping to SRL Milan. Samples were preserved in OM-S from a minimum of 4 days to a maximum of 22 days. Upon arrival at SRL Milan, OM-S was removed by centrifugation and one third of the sediment was inoculated into MGIT 960 tubes.

We obtained MTB growth in samples preserved in OM-S for as long as 22 days and these included samples with a low smear grade as well as smear negative ones. Among the samples preserved in OM-S, 42 (12.8%) were culture positive, 286 (86.9%) were culture negative and 1 (0.3%) was contaminated, while among the NALC/NaOH treated ones, 45 (13.7%) were culture positive, 241 (73.3%) negative and 43 (13%) were contaminated**.** Notably, the overall culture contamination rate of the OM-S treated samples was lower than 0.5% (0.3%), while that of NALC/NaOH treated ones was 13%. Among the 48 smear positive samples, the number of samples identified as MTB positive by liquid culture was 33 (68.8%) and 35 (72.9%) for the OM-S and the NALC/NaOH treatment groups respectively, while among the 281 smear negative samples, MTB was isolated in 9 (3.2%) and 10 (3.6%) of the OM-S and the NALC/NaOH groups respectively.

**Fig. 1:**
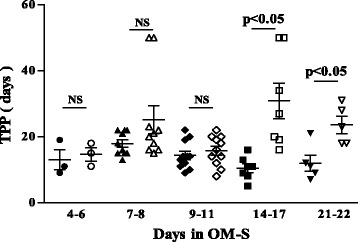
Comparison of time to culture positivity between OM-S preservation and NALC/NaOH decontamination, dependent on time in OM-S. Time to culture positivity (TPP) of the culture positive samples preserved in OM-S (empty symbols) or processed by NALC/NaOH method (full symbols). Error bars indicate SEM

We next evaluated the time to culture positivity (TPP) (i.e. the interval of days from the date of inoculation to the date of observable growth) of the culture positive samples preserved in OM-S and those decontaminated by NALC/NaOH. We found that OM-S treatment negatively affected the TPP with an average TTP of 22 days as compared to the 14,5 days of the NALC/NaOH treatment group (data not shown). This delay in growth was greater for samples with a low smear grade (i.e. 1+ and scanty) and it correlated to the total number of days of preservation in OM-S. The difference of TPP was significantly greater for samples that were preserved in OM-S for more than two weeks (Fig. 1).

A total of 36 (10.9%) samples were culture positive, 1 (0.3%) contaminated and 240 (73%) culture negative in both treatment groups, whereas 15 (4.5%) samples showed a discrepant culture result. In particular, 9 (2.7%) (6 smear positive and 3 smear negative) samples were culture positive in the NALC/NaOH treatment group but culture negative in the OM-S group, while 6 (1.8%) samples that were either contaminated (5 samples) or negative (1 sample) in the NALC/NaOH group, were culture positive upon processing with OM-S reagent (Table 1). Notably, the absence of growth in the OM-S treated samples did not correlate to the length of preservation in the reagent which ranged from 7 to 18 days nor to the bacterial load.

**Table 1:** Culture comparison between NALC/NaOH and OMNIgene-SPUTUM treatment groups

		NALC/NaOH treatment		
	Culture	Positive	Negative	Contaminated
OM-S treatment	Positive	36	1	5
	Negative	9	240	37
	Contaminated	0	0	1

Number of culture positive, culture negative and culture contaminated among samples in the NALC/NaOH or OMNIgene-SPUTUM (OM-S) treatment groups

### Performance of OM-S decontaminated specimens in MTBDRplus assay

We next assessed the capacity of OM-S reagent to preserve DNA stability for genotypic drug susceptibility testing by LPA using the GenoType MTBDR*plus* (Hain Lifescience, Nehren, Germany) assay. DNA was extracted from one third of the OM-S treated sediment (i.e. 0.5 ml) using the commercially available GenoLyse kit (Hain Lifescience). LPA was performed on all samples with a positive MTB culture regardless of the decontamination method used. Out of 51 specimens tested, 45 (88.2%) showed a complete hybridization profile, 3 (5.9%) samples only a partial profile with one or more target regions missing and 3 (5.9%) samples a negative result (i.e. TUB band negative and none of the target regions amplified). Notably, the samples with a negative or only partially available LPA result were either smear negative (3 samples) or had a low smear grade (1+), a data consistent with the lower sensitivity of this assay in smear negative samples. For the 9 OM-S culture negative- NALC/NaOH culture positive discrepant samples GenoTypeMTBDR*plus* revealed the presence of MTB in 8 out of 9 cases. This data indicates that while long-term storage of sputum samples in OM-S reagent has a negative effect on MTB viability, it does not affect the bacterial DNA stability even after prolonged preservation at room temperature.

## Discussion

This is the first study to assess the viability of MTB in clinical specimens preserved for a prolonged period of time in OM-S reagent in the absence of refrigeration.

Our findings show that OM-S is a valuable tool for the preservation of clinical samples for liquid culture at least up to three weeks of incubation at ambient temperature (20–25 °C) and it has good performance for the maintenance of sample integrity for molecular testing. Recent studies have shown OM-S compatibility with XperMTB/RIF testing [7]. Here we demonstrate that OM-S is also compatible with the DNA extraction kit GenoLyse and the GenoType MTBDR*plus* assay for rapid detection of resistance to first line anti-TB drugs. Importantly, while previous studies evaluated OM-S for the preservation of samples for relatively short times, up to 8 days for culture and 13 days for XpertMTB/RIF testing, we extended the testing time to up to 22 days to provide a more realistic simulation of in-field conditions.

Overall, OM-S characteristics are particularly relevant for settings with a weak sample transport and referral system or for hard to reach locations where clinical samples are usually batched before their shipping to a reference laboratory for testing. Notably, OM-S reagent offers an important advantage over cetyl-pyridinium chloride (CPC) a bacteriostatic substance used for the preservation of sputum for mycobacterial culture [8–11], by allowing the growth of the bacteria in both solid [4] and liquid media, while CPC treatment is not suitable for culture by Bactec MGIT 960 system [12].

In our study, OM-S effectively liquefied sputum samples and greatly eased the decontamination process halving the hands-on time for culture processing. Importantly, OM-S improved the quality of cultures by decreasing the contamination rate to less than 0.5%. However, contrary to a previous publication [4], we observed a delay of approximately one week in the time to culture positivity upon OM-S treatment as compared to NALC/NaOH standard method and a loss of MTB viability in 20% (9 out of 45) of the NALC/NaOH treated culture positive samples. This delay in bacterial growth and loss of viability are unlikely due the presence of residual chemistry in the OM-S treated sediments given that supernatants were carefully removed upon arrival to the laboratory, but rather to the high decontaminating power of OM-S reagent. This hypothesis is supported by the overall very low culture contamination rate for the OM-S treated samples and by the presence of MTB DNA in the great majority of OM-S negative/NALC/NaOH culture positive samples. Analytical studies should be performed at DNAGenotek to further improve OM-S composition to achieve a better balance between the reagent decontaminating power and the maintenance of MTB viability while at the same time minimizing the delay in culture turn-around times.

We acknowledge as a limitation of this study the fact that aliquots from the same sample were processed for culture at two different reference laboratories. The limited volume of the sputa and the heterogeneity of the samples did not allow for testing multiple aliquots at both testing sites. Importantly, the primary objective of this study was not the head to head comparison of two sample processing procedures but to evaluate the performance of OM-S for the long-term preservation of sputum specimens by mimicking conditions often encountered in low resources- high TB incidence countries.

## Conclusions

Overall, OM-S is a promising reagent for the storing and transport of clinical specimens in the absence of refrigeration allowing the maintenance of MTB viability and the preservation of MTB DNA integrity for extended period of times. These results have important implications for National TB programs by significantly reducing the costs related to sample storage and transport, by increasing the program capacity to reach populations living in settings with poor access to TB diagnostic facilities and ultimately by improving the management of TB patients by reducing the likelihood of providing multiple samples for TB testing.

## Abbreviations

CPC: Cetyl-pyridinium chloride
DST: Drug susceptibility testing
LPA: Line probe assay
MTB: *Mycobacterium tuberculosis*
NALC: N-acetyl L-cysteine
NRL: National Reference Laboratory
OM-S: OMNIgene-SPUTUM
SRL: Supranational Reference Laboratory
TAT: Turn-around-time
TB: Tuberculosis
TPP: Time to culture positivity
WHO: World Health Organization
